# *In vitro* and *in silico* analysis of imatinib analogues as anti-*Trypanosoma cruzi* drug candidates

**DOI:** 10.1017/S0031182023000057

**Published:** 2023-04

**Authors:** Luca S. F. Nesic de Freitas, Cristiane França da Silva, Sebastiano Intagliata, Emanuele Amata, Loredana Salerno, Maria de Nazaré Correia Soeiro

**Affiliations:** 1 Laboratório de Biologia Celular, Instituto Oswaldo Cruz, Fiocruz, RJ, Brazil; 2 Department of Drug and Health Sciences, Section of Medicinal Chemistry, University of Catania, Catania, Italy

**Keywords:** Chagas disease, drug repurposing, imatinib, imatinib analogues, *in vitro*, *in silico*

## Abstract

Chagas disease (CD) is a neglected tropical disease caused by the intracellular protozoan *Trypanosoma cruzi* that remains a serious public health issue affecting more than 6 million people worldwide. The available treatment includes 2 nitro derivatives, benznidazole (BZ) and nifurtimox, that lack in efficacy in the later chronic phase and when administered against the several naturally resistant parasite strains and present several side-effects, demanding new therapeutic options. One strategy is based on repurposing by testing drugs already used for other illness that may share similar targets. In this context, our previous data on imatinib (IMB) and derivatives motivated the screening of 8 new IMB analogues. Our findings showed that all except 1 were active against bloodstream trypomastigotes reaching drug concentration capable of inducing a 50% of parasite lysis (EC_50_) values < 12 *μ*m after 2 h while BZ was inactive. After 24 h, all derivatives were more potent than BZ, exhibiting EC_50_ values 1.5–5.5 times lower. Against intracellular forms, 7 out of 8 derivatives presented high activity, with EC_50_ values ≤ BZ. LS2/89 stood out as one of the most promising, reaching EC_90_ values of 1.68 and 4.9 *μ*m on intracellular and trypomastigote forms, respectively, with the best selectivity index (>60) towards the proliferative forms. Physicochemical parameters as well as the absorption, distribution, metabolism, excretion and toxicity properties were predicted to be acceptable and with good chance of a favourable oral bioavailability. The promising results motivate further studies such as *in vivo* and combinatory assays aiming to contribute for a novel safer and effective therapy for CD.

## Graphical abstract

**Figure:**
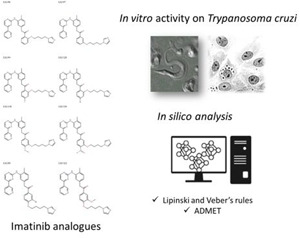

## Introduction

Chagas disease (CD) is a trypanosomiasis caused by the protozoan parasite *Trypanosoma cruzi*. It was discovered in 1909 by Dr Carlos Chagas (Chagas, 1909) and currently affects over 6 million people worldwide (WHO, 2022), mostly impoverished populations with little to no access to health care. Because of that, though CD only has ineffective and toxic available treatments, it receives little attention from most pharmaceutical industries for the development of safer and more active remedies. For these reasons, CD is classified as a neglected tropical disease (NTD) (DNDi, 2022).

This disease has 2 distinct phases: the acute phase, characterized by patent parasitaemia, often with non-specific symptoms or none. This phase usually lasts 4–8 weeks and, due to host immune response, the parasite burden is controlled but not extinguished, and most individuals move to the chronic phase. This second stage is characterized by a sub-patent and intermittent parasitaemia and is often asymptomatic as well. However, after years or decades, by still not well-known mechanisms, 30–40% of the infected people develop intestinal and/or cardiac pathologies (Pérez-Molina and Molina, 2018).

Currently, the treatment available is the one developed over 5 decades ago which uses the nitro derivatives benznidazole (BZ) and nifurtimox. However, these old drugs are inadequate in that they may induce severe side-effects, which may even make it necessary to interrupt the treatment and are lacking in efficacy when administered in the later stage of the disease as well as against naturally resistant strains of the parasite (Bermudez *et al*., 2016).

Since a new treatment is needed as soon as possible and, as a neglected disease, CD research lacks funding and manpower, drug development strategies which can reduce time and cost are greatly valued (Soeiro, 2022). One strategy which fits these criteria is drug repurposing, which tests pharmacological agents already used for the treatment of other illnesses and that share common mechanism of action and cellular targets. Once a promising candidate is identified, analogues of the original molecule are synthesized with the goal of optimizing the effect against this new disease (Ashburn and Thor, 2004; Ochiana *et al*., 2013).

Imatinib (IMB) is a tyrosine kinase inhibitor used in the treatment of cancers, such as chronic myeloid leukaemia, which was identified as a candidate for drug repurposing by our group (Simões-Silva *et al*., 2019). Presently, 8 new IMB analogues recently synthesized as novel anti-leukaemic drugs (Sorrenti *et al*., 2018) were tested for their *in vitro* trypanocidal activity and cytotoxicity compared to the original molecule, IMB, as well as the reference drug, BZ. In addition, the *in silico* profile was assessed while taking into account the drug-likeness of the IMB analogues.

## Materials and methods

### Compounds

**Fig. 1.**
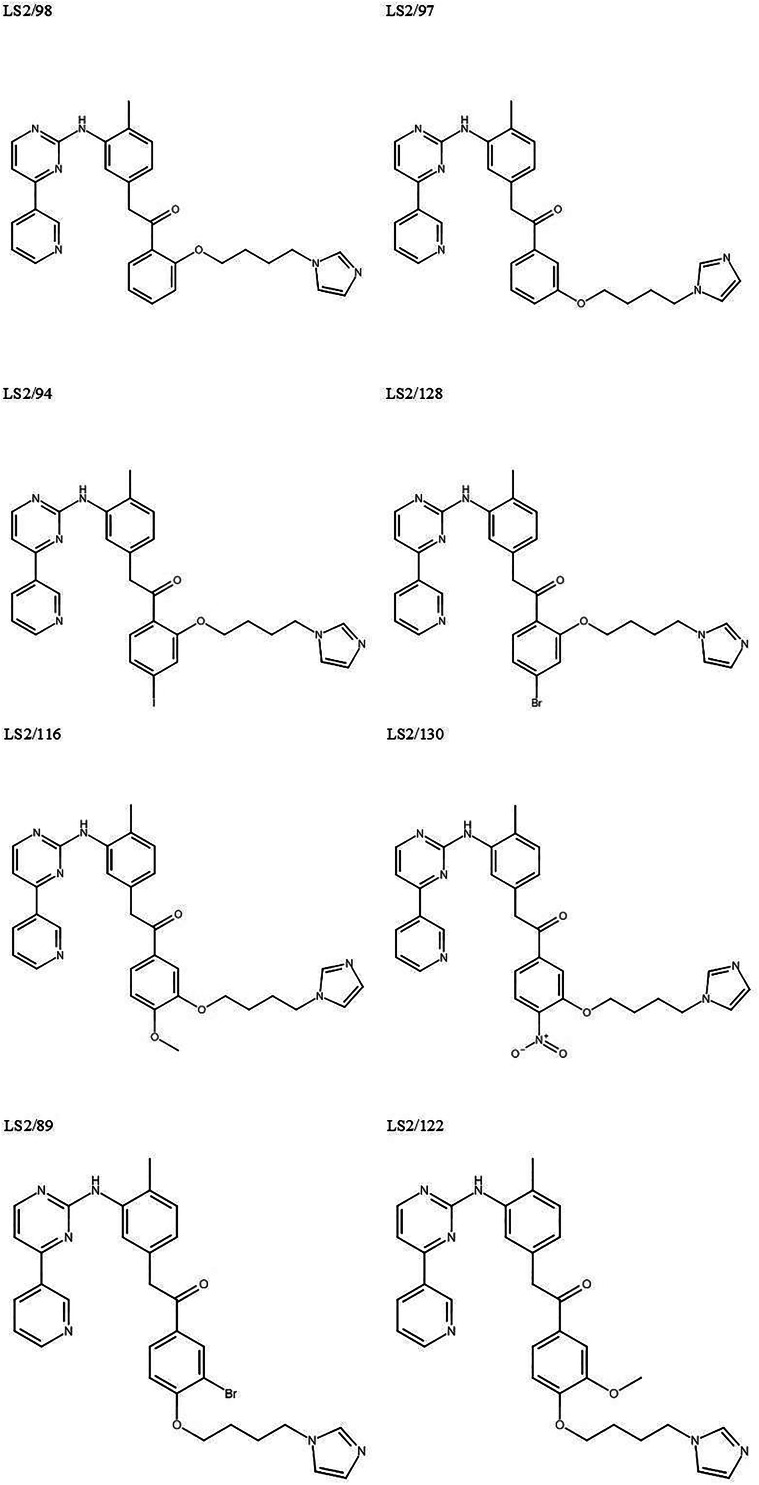
Molecular structure of the IMB analogues.

As depicted in Fig. 1, 8 IMB analogues were synthesized in the Department of Drug and Health Sciences of the University of Catania and kindly provided for *in vitro* testing. The purity of all final compounds was ≥95% as previously described (Sorrenti *et al*., 2018). BZ (LAFEPE, Brazil) was used as a reference drug for CD. Stock solutions of BZ and the tested compounds were prepared in 100% dimethyl sulphoxide (DMSO), with the final in-test concentration never exceeding 0.6% for *in vitro* experiments to avoid non-specific toxicity (Cardoso-Santos *et al*., 2022).

### Parasites

Bloodstream trypomastigotes (BT) of *T. cruzi* (Y strain, DTU II) were obtained from Swiss Webster mice at the peak of parasitaemia as reported (Meirelles *et al*., 1986). The trypomastigote forms of Tulahuen strain (DTU VI) expressing the *Escherichia coli β*-galactosidase gene were collected from the supernatant of L929 cell cultures previously infected (host:parasite cell ratio 10:1) (Romanha *et al*., 2010). For both strains, purified parasites were added to Roswell Park Memorial Institute (RPMI) 1640 medium supplemented with 5% fetal bovine serum (FBS) to perform assays at 37°C in 5% CO_2_. All animal studies were carried out in strict accordance with the guidelines established by the FIOCRUZ Committee of Ethics for the Use of Animals (CEUA L038-2017).

### Mammalian cell cultures

L929 cell lines were routinely maintained through weekly dissociation with 0.01% trypsin solution followed by plating with 4 × 10^3^ cells per well in 96-well microplates and sustained at 37°C in RPMI 1640 medium (Sigma-Aldrich, Saint Louis, MO) (Romanha *et al*., 2010).

### Cytotoxicity assay

L929 culture cells were seeded in 96-well plates with 4 × 10^3^ cells per well and sustained in RPMI medium supplemented with 10% FBS. The compounds were added (0–400 *μ*m, serially diluted 1:2) and the non-infected cultures incubated for 96 h at 37°C/5% CO_2_. Then, their viability was assessed by Alamar Blue reagent following the manufacturer's specifications. Controls were carried out with parasites kept under the same conditions in the absence of the compounds. The data were expressed by the drug concentration capable of inducing a 50% loss of host cell viability (LC_50_) value which represents the concentration capable of inducing a 50% loss of cellular viability (Romanha *et al*., 2010).

### *In vitro* activity against bloodstream trypomastigotes (Y strain)

Trypomastigote forms were incubated in 96-well microplates. Briefly, 100 *μ*L of a BT suspension (in RPMI medium + 5% FBS) containing 10^7^ parasites mL^−1^ was added to the same volume of each compound diluted in RPMI + 5% FBS at twice of the desired final concentration (0–50 *μ*m, serially diluted 1:2). After 2 and 24 h at 37°C, the number of live parasites was determined by light microscope quantification using a Neubauer chamber. Controls were carried out with parasites kept under the same conditions in the absence of the compounds. BZ was run in parallel. The anti-parasitic activity of the compounds was expressed by the EC_50_ and EC_90_ values after 2 and 24 h of incubation, which represent the concentration capable of inducing a 50 and 90% of parasite lysis, respectively (Bouton *et al*., 2021; Lin *et al*., 2021).

### *In vitro* activity against intracellular forms of *T. cruzi* (Tulahuen strain transfected with *β*-galactosidase gene, DTU VI)

L929 cells were infected with trypomastigotes obtained from the supernatant of infected cultures. After 2 h of interaction (10 parasites per host cell), the parasites which were not internalized were removed by replacing the RPMI medium. After 48 h of incubation, the compounds were added to the infected cultures (0–10 *μ*m, serially diluted 1:2) and the cultures incubated for 96 h at 37°C/5% CO_2_. BZ and DMSO (solvent used for the compounds) were run in parallel as positive and negative controls, respectively. After the elapsed time, 50 *μ*L per well of chlorophenol red-*β*-D-galactopyranoside was added and a reading was done in a spectrophotometer at 570 nm (Romanha *et al*., 2010). The activity of the compounds was expressed by the EC_50_ and EC_90_ values, which represent the concentration capable of inducing a 50 and 90% loss of viability in the parasites, respectively (Romanha *et al*., 2010).

### Data analysis and EC_50_, EC_90_ and IC_50_ calculation

EC_50_, EC_90_ and LC_50_ calculation, as well as the 95% confidence interval presented in lieu of standard deviation, was performed by Prism Graphpad Version 9.1.0 using non-linear regression with the data obtained in at least 2 assays in triplicate.

### *In silico* studies

*In silico* analysis was performed using the SwissADME platform, developed by the Swiss Institute of Bioinformatics. This platform has 6 different algorithms for estimating the partition coefficient, each with its pros and cons and as such the iLogP model was selected as it presented a better estimate with respect to the original molecule (IMB) and was thus conjectured to be a suitable model for its analogues (Daina *et al*., 2017). Lipinski's rules of 5 and Veber's rules violations were assessed as perspectives in *in silico* analysis for estimating drug-likeness and oral bioavailability (Veber *et al*., 2002; McKerrow and Lipinski, 2017).

## Results

Our first approach was the determination of the cytotoxicity of the studied compounds as depicted in Table 1. The data show LS2/94 displayed a non-toxic profile like BZ, exhibiting an LC_50_ value > 400 *μ*m. The other compound presented a mild toxicity profile with LC_50_ values ranging from 5 to 19 *μ*m after 96 h of incubation with L929 cell cultures (Table 1). Next, the anti-parasitic activity against intracellular forms (Tulahuen strain, DTU VI) was evaluated (Table 1). Our findings demonstrated that, except for LS2/94 (EC_50_ > 10 *μ*m), all analogues were active, presenting similar or even higher potency than BZ (EC_50_ = 4.1 *μ*m). Among them, LS2/89 was the most potent (EC_50_ = 0.19 *μ*m), being about 22 times more active than the reference drug. LS2/89 also presented the greatest selectivity index (SI = 65) over L929 host cells. In addition, when compared to the parental compound (IMB, EC_50_ = 24.8 *μ*m), all analogues were more active, displaying potency from 5 to 132 times higher. Also, most of the test compounds (6 out of 8) were more selective than IMB (SI = 1.5), with selectivity indexes ranging from 3.49 to 64.74 (Table 1). Interestingly, 2 compounds reached EC_90_ values < 10 *μ*m, and again, LS2/89 gave an outstanding result, achieving the lowest value (1.6 *μ*m) and being about 4.6-fold more effective than BZ that has EC_50_ = 7.79 *μ*m (Table 1).

**Table 1.** *In vitro* trypanocidal activity (EC_50_, EC_90_ values in *μ*M, with 95% confidence interval) of the imatinib analogues, as well as benznidazole (BZ) and imatinib (IMB) (EC_50_ values in *μ*M ± s.d.) against intracellular forms of *Trypanosoma cruzi*, cytotoxicity on L929 cell lines (LC_50_ in *μ*M) as well as the respective selectivity index over L929 host cells (SI = LC_50_/EC_50_)

Compound	Toxicity against L929 cells	Activity against intracellular forms of the parasite		Selectivity index (IC_50_/EC_50_)
	LC_50_ (*μ*M)	EC_50_ (*μ*M)	EC_90_ (*μ*M)	
LS2/98	12.20 (2.64–56.34)	3.31 (2.05–5.34)	>10	3.93
LS2/97	19.26 (6.31–58.80)	1.30 (0.75–2.26)	>10	14.81
LS2/94	>400	>10	>10	ND
LS2/128	17.92 (9.92–32.34)	4.25 (2.94–6.13)	>10	4.22
LS2/116	5.13 (1.21–21.79)	1.47 (1.01–2.13)	>10	3.49
LS2/130	5.08 (1.16–22.25)	1.05 (0.49–2.26)	9.44 (4.39–20.31)	4.84
LS2/89	12.30 (4.26–35.50)	0.19 (0.10–0.33)	1.68 (0.95–2.98)	64.74
LS2/122	5.44 (1.32–22.41)	4.47 (2.84–7.05)	>10	1.22
BZ	>400	4.1 ± 1.3	ND	>97.6
IMB	38.3 ± 0.2	24.8 ± 7.4	ND	1.5

ND, not determined

BZ and IMB data from Simões-Silva *et al*. (2019).

Next, the activity of IMB effect was further analysed upon different parasite strain and forms. Thus, when assayed against BT (Y strain, DTU II), all test compounds were highly active, with 7 out of 8 also presenting fast-killer profile, achieving EC_50_ and EC_90_ values lower than 12 and 30 *μ*m, respectively, after only 2 h of incubation while BZ is completely inactive after this short period of drug exposure (Table 2). The test compounds showed EC_50_ between 4 and 12 *μ*m whereas the reference drug does not reach its EC_50_ up to 50 *μ*m. Once again LS2/89 was among the most potent analogues with an EC_50_ of 2.67 *μ*m after 24 h of incubation, being over 5 and 12 times more potent than BZ (14.4 *μ*m) and IMB (33.6 *μ*m), respectively. With respect to the EC_90_, a relevant parameter when considering the possibility of total elimination of parasites, all compounds were able to reach more than 90% death rates of the parasites after 24 h (Table 2) at a lower concentration than BZ (22.8 *μ*m).

**Table 2.** *In vitro* trypanocidal activity of the imatinib analogues (EC_50_ and EC_90_ values in *μ*M, with 95% confidence interval) as well as benznidazole and imatinib (EC_50_ and EC_90_ values in *μ*M ± s.d.) against bloodstream trypomastigote forms of *Trypanosoma cruzi* (Y strain) after 2 and 24 h of incubation

Compound	Activity against bloodstream trypomastigotes (*μ*M)			
	2 h		24 h	
	EC_50_	EC_90_	EC_50_	EC_90_
LS2/98	>50	>50	9.59 (7.22–12.75)	21.99 (19.58–24.69)
LS2/97	7.84 (5.46–11.26)	11.44 (10.86–12.06)	4.72 (3.29–6.77)	8.52 (7.26–10.01)
LS2/94	3.97 (2.54–6.20)	5.05 (2.35–10.85)	2.60 (1.84–3.67)	4.48 (3.95–5.08)
LS2/128	5.93 (4.22–8.32)	8.93 (7.24–11.00)	4.12 (2.68–6.33)	5.69 (5.42–5.97)
LS2/116	12.00 (8.26–17.44)	28.11 (13.40–58.97)	3.89 (2.74–5.53)	7.09 (6.21–8.09)
LS2/130	5.69 (4.06–7.97)	7.31 (6.19–8.63)	3.40 (2.32–4.99)	4.80 (4.01–5.75)
LS2/89	4.83 (3.39–6.90)	8.12 (7.41–8.89)	2.67 (1.92–3.71)	4.93 (4.08–5.94)
LS2/122	11.09 (7.95–15.47)	22.09 (14.05–34.75)	3.27 (2.30–4.65)	5.34 (4.84–5.91)
BZ	>50	>50	14.4 ± 3.4	22.8 ± 7.8
IMB	>50	>50	47.1 ± 9.5	>50

BZ and IMB data from Simões-Silva *et al*. (2019).

Regarding *in silico* physicochemical analysis depicted in Table 3, the analogues only slightly violated Lipinski's rule with respect to molar mass, exceeding the cut-off of 500 g mol^−1^ but with no further violations. As for Veber's rules, the compounds exceeded 10 rotatable bonds though only LS2/130 exceeded the 140 Å^2^ cut-off for total polar surface area. Neither BZ nor IMB violate neither Lipinski's nor Veber's rules (Table 3). Concerning absorption, distribution, metabolism, excretion, and toxicity (ADMET) properties (Table 4), all test compounds were predicted to have low permeability on gastrointestinal cells, are not likely to cross through the blood–brain barrier neither metabolized by CYP1A2 similarly to BZ and IMB (Table 4).

**Table 3.** *In silico* physicochemical parameters of the analogues as well as IMB and BZ

Property	Reference value	LS2/98	LS2/97	LS2/94	LS2/128	LS2/116	LS2/130	LS2/89	LS2/122	BZ	IMB
Molar mass (g mol^−1^)	≤500 (Lipinski)	519.6	519.6	645.49	598.49	549.62	564.59	598.49	549.62	260.25	493.6
iLogP	≤5 (Lipinski)	3.69	3.9	4.11	4.18	3.59	3.39	4.17	3.79	1.15	4.04
Number of hydrogen bond acceptors	≤5 (Lipinski)	2	2	2	2	2	2	2	2	1	2
Number of hydrogen bond donors	≤10 (Lipinski)	6	6	6	6	7	8	6	7	4	6
Total polar surface area (Å^2^)	≤140 (Veber)	106.85	106.85	106.85	106.85	116.08	152.67	106.85	116.08	92.74	86.28
Number of rotatable bonds	≤10 (Veber)	12	12	12	12	13	13	12	13	6	8

**Table 4.** *In silico* ADMET profile of the imatinib analogues, imatinib (IMB) and benznidazole (Bz)

Compound	LS2/98	LS2/97	LS2/94	LS2/128	LS2/116	LS2/130	LS2/89	LS2/122	BZ	IMB
Gastro intestinal absorption	Low	Low	Low	Low	Low	Low	Low	Low	High	High
Blood–brain barrier permeation	No	No	No	No	No	No	No	No	No	No
P-gp substrate	Yes	Yes	No	No	Yes	No	No	Yes	No	Yes
CYP1A2 inhibitor	No	No	No	No	No	No	No	No	No	No
CYP2C19 inhibitor	Yes	Yes	Yes	Yes	Yes	Yes	Yes	Yes	No	Yes
CYP2C9 inhibitor	Yes	Yes	Yes	Yes	Yes	Yes	Yes	Yes	No	Yes
CYP2D6 inhibitor	Yes	Yes	Yes	Yes	Yes	No	Yes	Yes	No	Yes
CYP3A4 inhibitor	Yes	Yes	Yes	Yes	Yes	Yes	Yes	Yes	No	Yes

## Discussion

CD kills annually more people in Latin America than any other parasitic disease, but despite this scenario, its treatment is still based on 2 old and toxic anti-parasitic nitro derivative drugs which still represent a big challenge requiring new therapeutic alternatives (Soeiro, 2022). Repositioning represents a faster and cheaper strategy, and our previous data on IMB and 14 novel derivatives gave promising *in vitro* effect on *T. cruzi*. IMB was moderately active against different strains and forms of the protozoan and 1 analogue was as potent as BZ (Simões-Silva *et al*., 2019). These findings encouraged us to presently investigate the effect of 8 novel IMB derivatives against both forms relevant for mammalian infection: intracellular forms and BT besides considering the aspects of mammalian host toxicity *in vitro*. Our present assays show that these IMB analogues possess significant trypanocidal activity, greatly surpassing the original molecule (IMB) as well as the reference drug (BZ) while retaining moderate toxicity (Simões-Silva *et al*., 2019). The *in silico* profile, while not entirely compliant with Lipinski's and Veber's rules, was acceptable and showed a promising degree of drug-likeness and a good chance of a favourable oral bioavailability. Though Lipinski's rules do not necessarily exclude a drug candidate, it is a well-established and a first-step useful analysis of drug-likeness properties. Regarding the Veber's rules, the fact that all of these compounds are at least partially compliant to these sets of parameters represents another favourable drug-likeness indicative in addition to their promising anti-parasitic effect *in vitro* aiming to contribute for the identification of novel therapeutic alternatives to this NTD that affects more than 6 million individuals.

Among the test analogues, LS2/94 was not toxic to mammalian cells up to 400 *μ*m and was the only one that was not active up to 10 *μ*m when assayed upon intracellular forms of the parasite, though it presented a great trypanocidal profile against BT (EC_50_ values of 3.9 and 2.6 *μ*m after 2 and 24 h of drug exposure, respectively). The intracellular amastigotes are the proliferative forms present in the host mammalian cells and are those that have a high active metabolism. LS2/94 has a remarkable activity against trypomastigotes (highly infective but not proliferative form) being almost 5.5 times more potent than BZ. After 24 h of incubation, LS2/94 reached an outstanding EC_90_ value of 3.46 *μ*m that is at least 6 times lower than the corresponding value for the reference drug for CD (BZ). In fact, all test compounds achieved 100% of BT lysis after 24 h of incubation. This characteristic is largely desirable since low metabolic forms such as trypomastigotes as well as dormant/persister parasites have been related to therapeutic failures (Maclean *et al*., 2018). According to the current literature, persister-like cells may tolerate high drug pressure for long periods of exposure, being able to resume growth after drug withdrawal (Soeiro, 2022). Thus, compounds that have the ability to act towards non-metabolic forms are very promising, especially in combinatory use with other anti-*T. cruzi* drug candidates highly active against intracellular forms such as BZ and nucleoside derivatives (Bouton *et al*., 2021; Lin *et al*., 2021; Cardoso-Santo *et al*., 2022). A future combination approach of this analogue with molecules active against intracellular forms could yield positive results. In this sense, we found that most of the test IMB analogues are very potent against intracellular forms and one of them which stands out is LS2/89. This analogue presented a fast-killer profile against BT and a high activity against the intracellular proliferative forms (EC_50_ and EC_90_ values of 0.19 and 1.68 *μ*m, respectively), leading to the greatest selectivity index (SI = 65) among the IMB analogues tested. These promising data classify LS2/89 as a hit anti-*T. cruzi* compound (e.g. EC_50_ < 5 *μ*m, reaching max activity > 95%, selectivity > 10) as recommended (Kratz *et al*., 2021; Soeiro, 2022). Then, the bulk of our results support further investigation of IMB analogues as anti-*T. cruzi* agents and represent a successful repurposing approach to be further tested in *in vivo* assays alone and in combination with BZ aiming to contribute for drug discovery programmes of novel CD therapies.

## Data Availability

The authors confirm that all the data supporting the findings presented in the present study are available in the manuscript. Raw data are available from the corresponding and last author.
